# Functional Crosstalk between the PP2A and SUMO Pathways Revealed by Analysis of STUbL Suppressor, *razor 1-1*

**DOI:** 10.1371/journal.pgen.1006165

**Published:** 2016-07-11

**Authors:** Minghua Nie, Emily Arner, John Prudden, Lana Schaffer, Steven Head, Michael N. Boddy

**Affiliations:** 1 Department of Cell and Molecular Biology, The Scripps Research Institute, La Jolla, California, United States of America; 2 Microarray and Next Generation Sequencing Core Facility, The Scripps Research Institute, La Jolla, California, United States of America; UCSD, UNITED STATES

## Abstract

Posttranslational modifications (PTMs) provide dynamic regulation of the cellular proteome, which is critical for both normal cell growth and for orchestrating rapid responses to environmental stresses, e.g. genotoxins. Key PTMs include ubiquitin, the Small Ubiquitin-like MOdifier SUMO, and phosphorylation. Recently, SUMO-targeted ubiquitin ligases (STUbLs) were found to integrate signaling through the SUMO and ubiquitin pathways. In general, STUbLs are recruited to target proteins decorated with poly-SUMO chains to ubiquitinate them and drive either their extraction from protein complexes, and/or their degradation at the proteasome. In fission yeast, reducing or preventing the formation of SUMO chains can circumvent the essential and DNA damage response functions of STUbL. This result indicates that whilst some STUbL "targets" have been identified, the crucial function of STUbL is to antagonize SUMO chain formation. Herein, by screening for additional STUbL suppressors, we reveal crosstalk between the serine/threonine phosphatase PP2A-Pab1^B55^ and the SUMO pathway. A hypomorphic Pab1^B55^ mutant not only suppresses STUbL dysfunction, but also mitigates the phenotypes associated with deletion of the SUMO protease Ulp2, or mutation of the STUbL cofactor Rad60. Together, our results reveal a novel role for PP2A-Pab1^B55^ in modulating SUMO pathway output, acting in parallel to known critical regulators of SUMOylation homeostasis. Given the broad evolutionary functional conservation of the PP2A and SUMO pathways, our results could be relevant to the ongoing attempts to therapeutically target these factors.

## Author Summary

Posttranslational modifiers (PTMs) orchestrate the proteins and processes that control genome stability and cell growth. Accordingly, deregulation of PTMs causes disease, but can also be harnessed therapeutically. Crosstalk between PTMs is widespread, and acts to increase specificity and selectivity in signal transduction. Such crosstalk exists between two major PTMs, SUMO and ubiquitin, wherein a SUMO-targeted ubiquitin ligase (STUbL) can additionally mark SUMO-modified proteins with ubiquitin. Thereby, STUbL generates a hybrid SUMO-ubiquitin signal that is recognized by selective effectors, which can extract proteins from complexes and/or direct their degradation at the proteasome. STUbL function is critical to maintain genome stability, and it also mediates the therapeutic effects of arsenic trioxide in leukemia treatment. Therefore, a full appreciation of STUbL regulation and integration with other PTMs is warranted. Unexpectedly, we find that reduced activity of PP2A, a major cellular phosphatase, compensates for STUbL inactivation. Our results indicate that PP2A-regulated phosphorylation reduces the SUMO chain output of the SUMO pathway, thus reducing cellular dependency on STUbL and the functionally related factors Ulp2 and Rad60. Our data not only reveal a striking level of plasticity in signaling through certain PTMs, but also highlight potential "escape" mechanisms for SUMO pathway-based therapies.

## Introduction

Posttranslational modification (PTM) of the proteome drives most aspects of cell growth including cell cycle transitions, DNA replication, and DNA repair. Accordingly, deregulation of key PTMs such as phosphorylation, SUMOylation and ubiquitylation causes cell cycle defects, genome instability, and malignant transformation or cell death [1]. Crosstalk between PTMs in signal transduction is widespread [2], and has recently come to the fore in the SUMO and ubiquitin field. SUMO and ubiquitin are small protein PTMs that are covalently attached to target proteins via similar enzymatic cascades of E1 activating, E2 conjugating enzymes, and E3 ligases [3].

Both modifiers can form chains, with ubiquitin chains of different topologies supporting functions that range from proteolysis to protein recruitment [1, 3]. In contrast, physiological role(s) of SUMO chains are poorly defined, and blocking their formation has no discernible impact on fission yeast viability or genotoxin resistance [4]. In budding yeast, SUMO chain-deficient mutants exhibit reduced sporulation following meiosis, and an apparently pleiotropic impact on chromatin organization, transcription and genotoxin sensitivity [5, 6]. However, an earlier study on various SUMO chain mutants in budding yeast, with the exception of a drastic SUMO all K to R mutant, found no overt genotoxin sensitivities or growth defects [7]. Thus, any physiological requirement for SUMO chains is subtle.

In contrast to any positive roles, SUMO chains that accumulate in the absence of the desumoylating enzyme Ulp2 cause severe cell growth defects, genome instability, and genotoxin sensitivity [4, 7]. Accordingly, a SUMO^KtoR^ mutant that reduces SUMO chain formation rescues the phenotypes of *ulp2∆* fission and budding yeast [4, 7]. An accumulation of SUMO chains also causes the extreme genome instability and cell cycle phenotypes of fission yeast that lack the SUMO-targeted E3 ubiquitin ligase (STUbL) Slx8-Rfp1 [4, 8].

STUbLs bind SUMO chains through their amino-terminal tandem SUMO interaction motifs (SIMs) and ubiquitinate them using their carboxy-terminal RING domains [9–12]. Thereby, SUMO-ubiquitin hybrid chains are generated that can selectively recruit effector proteins with dual SUMO and ubiquitin binding domains. These effector proteins can either support further signaling e.g. Rap80 in the DNA damage response, or drive extraction of target proteins from complexes and/or their proteasomal degradation e.g. Cdc48-Ufd1-Npl4 [13]. Interestingly, inhibiting STUbL activity in both fission and budding yeast rescues *ulp2∆* cell phenotypes to a similar extent as blocking SUMO chain formation, despite an overall increase in SUMO chains [14, 15]. Therefore, unscheduled STUbL activity on SUMO-chain modified proteins, rather than the SUMO chains themselves, is toxic to *ulp2∆* cells.

From the above, it is clear that SUMO pathway homeostasis is critical to multiple processes that impact genome stability and cell growth. Moreover, the SUMO pathway and associated factors, like STUbL, are important therapeutic targets in cancer and other diseases (e.g. [16–18]). Therefore, the identification of activities that modulate SUMO pathway outputs is of fundamental importance. To this end, we exploited the lethality of the STUbL mutant *slx8-29* to screen for spontaneous suppressors, which resulted in the identification of a number of what we called "*razors*" due to their STUbL suppression. Here, we report the identification and characterization of one such *razor*, *rzr1-1*, which reveals novel functional crosstalk between the protein phosphatase PP2A and the SUMO pathway.

## Results

### *Rzr1-1* (Razor) a Suppressor of *slx8-29* (STUbL)

Hypomorphic mutants of the fission yeast STUbL Slx8 (e.g. *slx8-29*) are hypersensitive to a broad spectrum of genotoxins [8, 19–22]. When grown at semi-permissive temperature in the presence of genotoxins, spontaneous suppressors of *slx8-29* appear with a frequency of ~10^−7^. Here we present the identification and characterization of one such *slx8-29* suppressor, *rzr1-1*.

**Fig 1:**
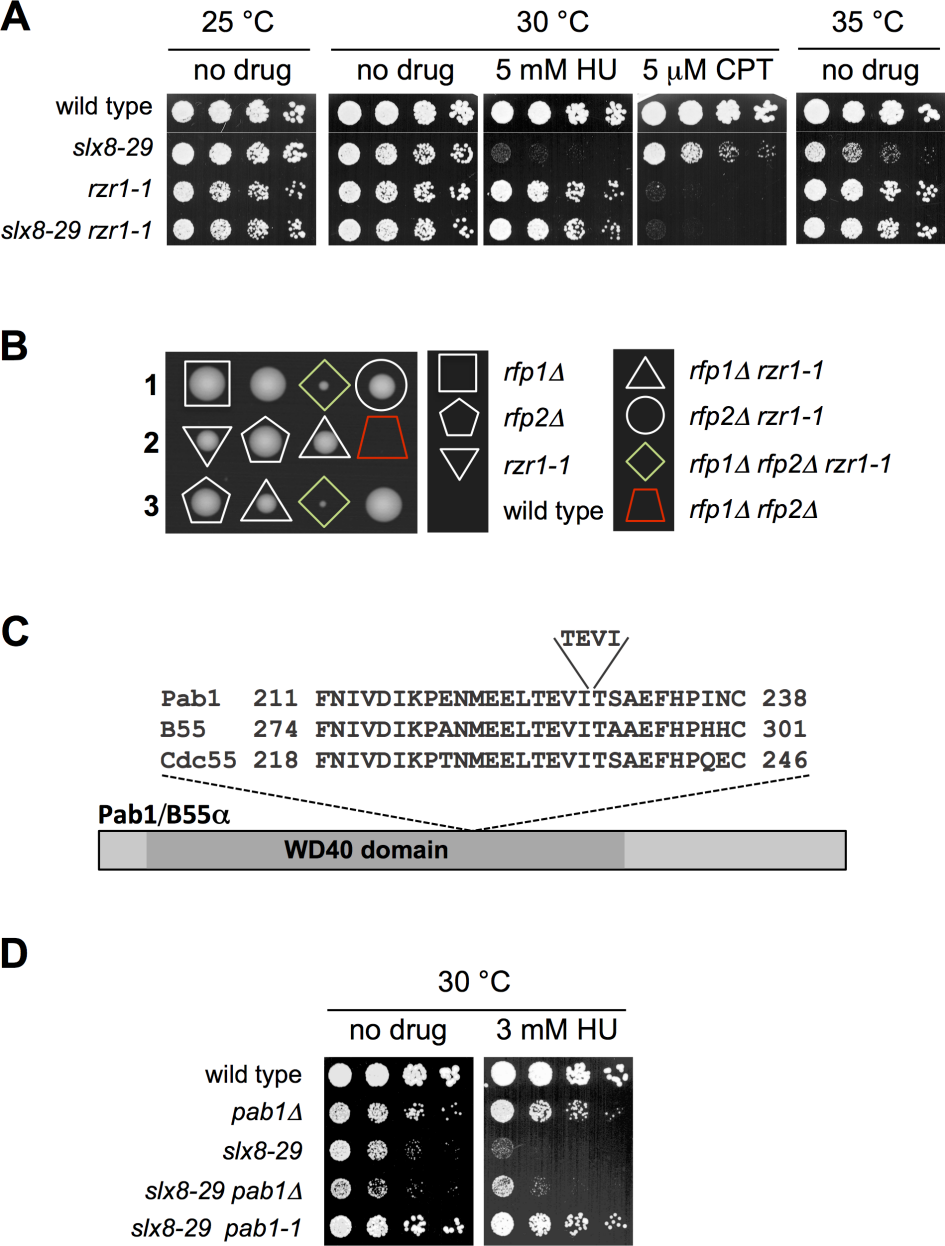
*Rzr1-1* (Razor) a Suppressor of *slx8-29* (STUbL). *A* and *D*, dilution series of the indicated strains were spotted onto YES plates, with or without drugs (HU: hydroxyurea; CPT: camptothecin) at the indicated concentrations and temperatures. *B*, three representative tetrad dissections are shown from a genetic cross between *rfp1Δ* and *rfp2Δ rzr1-1* cells. *C*, a schematic representation of the insertion mutation of *rzr1-1* in the conserved WD40 domain in the *pab1* locus. The indicated amino acids of the Bα subunits of PP2A in fission yeast (Pab1), human (B55) and budding yeast (Cdc55) are aligned, highlighting the high degree of sequence conservation across species. The "TEVI" insertion occurs at the end of a loop region between β3D and β4A in the seven-bladed β-propeller domain of Bα [24].

Backcrossing the *slx8-29 rzr1-1* strain to wild-type confirmed that *rzr1-1* is an extragenic suppressor of *slx8-29*. Testing the strains derived from the backcross for sensitivity to hydroxyurea (HU) and camptothecin (CPT) revealed potent suppression of the HU and temperature sensitivity of *slx8-29* by *rzr1-1* (**Fig 1A**). Interestingly however, *rzr1-1* cells are more sensitive to CPT than *slx8-29*, so do not afford a growth benefit in this condition.

Having confirmed suppression of *slx8-29* by *rzr1-1*, we then asked if *rzr1-1* could bypass the essential functions of STUbL. The RING finger proteins Rfp1 and Rfp2 form independent heterodimers with Slx8 to constitute STUbL activity [22]. Notably, whereas *rfp1∆ rfp2∆* cells are inviable [22], *rfp1∆ rfp2∆ rzr1-1* triple mutant cells are able to form small colonies (**Fig 1B**). Therefore, consistent with the rescue of *slx8*-29, the *rzr1-1* mutation strongly reduces cellular dependence on STUbL activity.

Classical genetic approaches to identify *rzr1-1* were unsuccessful, so we re-sequenced the genomes of the backcrossed *slx8-29 rzr1-1*, *slx8-29*, and *rzr1-1* strains. This analysis revealed that *rzr1-1* is a duplicated sequence in the gene encoding Pab1 (**Fig 1C**), the PR55 (Cdc55) regulatory B subunit of protein phosphatase 2A (PP2A, [23]). The insertion occurs in the highly conserved beta-propeller or WD40 domain of Pab1, which makes contact with the catalytic subunit of the PP2A holoenzyme, and is thought to contribute to substrate selection (**Fig 1C**, [24]). The duplication retains the reading frame of Pab1, and so generates a hypomorphic allele, which we will refer to as *pab1-1* from here on, rather than a null. Indeed, whereas *pab1-1* (*rzr1-1*) strongly suppresses *slx8-29*, a full deletion of Pab1 (*pab1∆*) causes poor growth with morphogenesis defects [23, 25] and provides only a marginal rescue of the temperature and HU sensitivity of *slx8-29* (**Fig 1D**).

### Pab1 Dosage Is Inversely Correlated with Growth of *slx8-29* Cells

**Fig 2:**
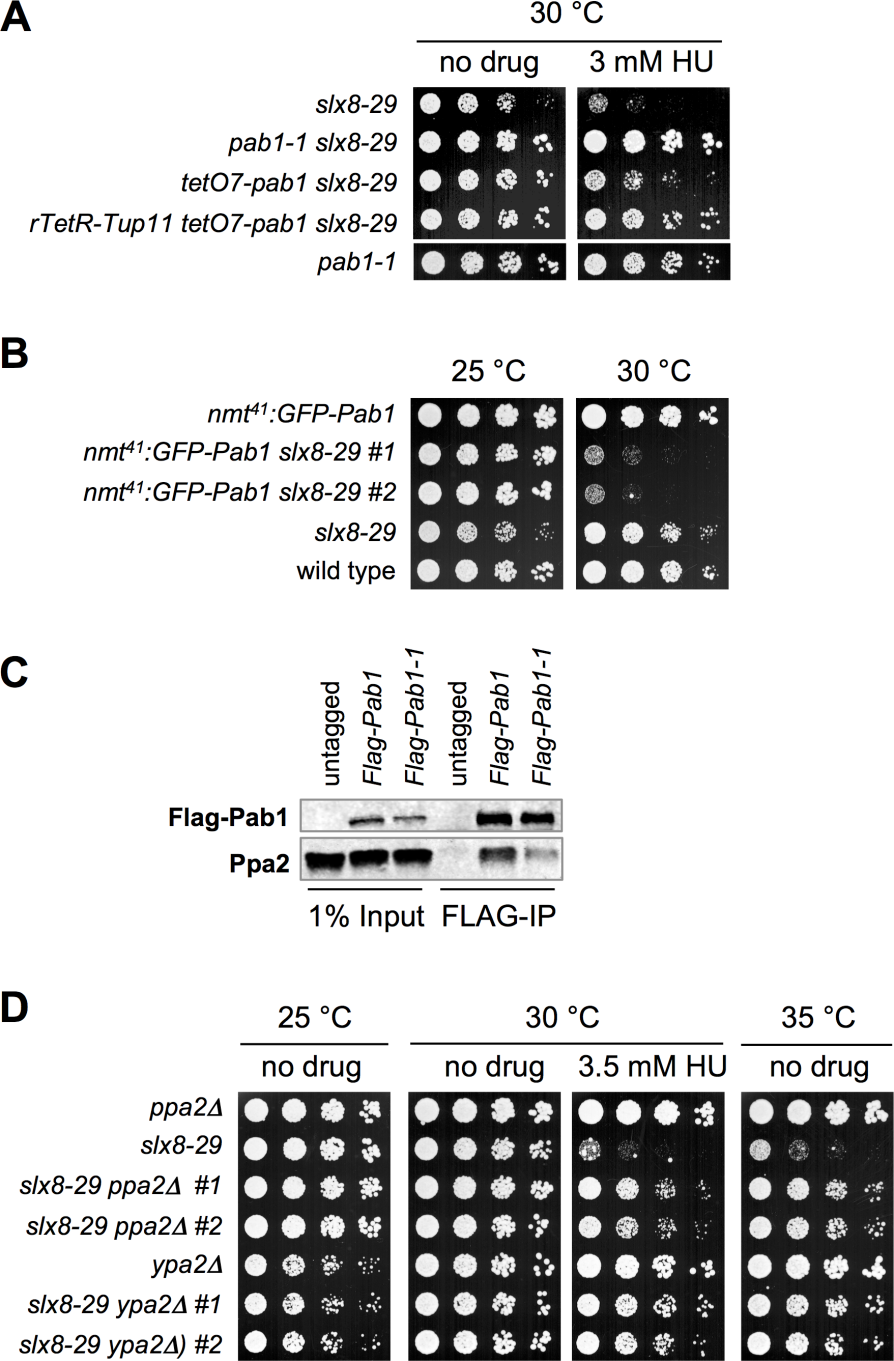
Pab1 Dosage Is Inversely Correlated with Growth of *slx8-29* Cells. *A*, *B and D*, dilution series of the indicated strains were spotted onto plates, with or without HU at the indicated concentrations and temperatures. The plates in *A* were YES also containing 5 μg/ml of anhydrotetracycline (ahTet), in *B* were minimum medium (EMM-LUAH) without thiamine (-B1) to induce the expression of GFP-Pab1 from the nmt41 promoter, and in *D* were YES without or with 3.5 mM HU. *C*, FLAG-IP of FLAG-tagged wild type Pab1 (lane 2, 5) or mutant Pab1-1 (lane 3, 6). Cells expressing untagged Pab1 (lane 1, 4) were used as control. 1% input and 20% of the FLAG-IP were analyzed by Western blotting with mouse-anti-FLAG, rabbit-anti-Ppa2 (Sunrise Science Products), and IRDye-conjugated anti-mouse^680^, anti-rabbit^800^ (Licor).

The *pab1-1* mutation lies in the beta-propeller region of Pab1 that is highly conserved within the B55 family, and could thus affect its association with the PP2A holoenzyme and/or substrates (**Fig 1C**, [24]). We therefore tested if *slx8-29* cells were sensitive to the dosage of wild-type Pab1. We replaced the endogenous promoter of Pab1 with a tetracycline-regulated promoter in cells that also express or not the TetR repressor [26], and assayed the effect on *slx8-29* cells. As for *pab1-1*, we found that reduced expression of wild-type Pab1 also rescued the HU sensitivity of *slx8-29* cells, especially in the presence of the TetR repressor (**Fig 2A**). In addition, overexpression of Pab1 from an attenuated *nmt* promoter (*nmt41*, [25, 27]) strongly impaired the growth of *slx8-29* but not wild-type cells (**Fig 2B**).

Given that Pab1 dosage is inversely correlated with the growth of *slx8-29*, we tested if the *pab1-1* mutation affects Pab1 protein levels. Western analysis indicates that the *pab1-1* mutation indeed destabilizes Pab1 and furthermore, appears to weaken its association with the PP2A catalytic subunit Ppa2 (**Fig 2C**). Together, these data suggest that *pab1-1* suppresses *slx8-29* phenotypes through a general reduction of PP2A-Pab1 activity. Consistent with this interpretation, deleting the catalytic subunit Ppa2 or the phosphotyrosyl phosphatase PP2A activator Ypa2 [28], also strongly suppresses the temperature and HU sensitivity of *slx8-29* cells (**Fig 2D**). Overall, our data reveal that PP2A-Pab1 activity drives the pathological effects of *slx8-29*, and that *pab1-1* likely provides an optimum general reduction in PP2A-Pab1 activity that allows cells to tolerate compromised STUbL function.

### *Pab1-1* Is Not a General Suppressor of DNA Repair and Replication Stress Defects

**Fig 3:**
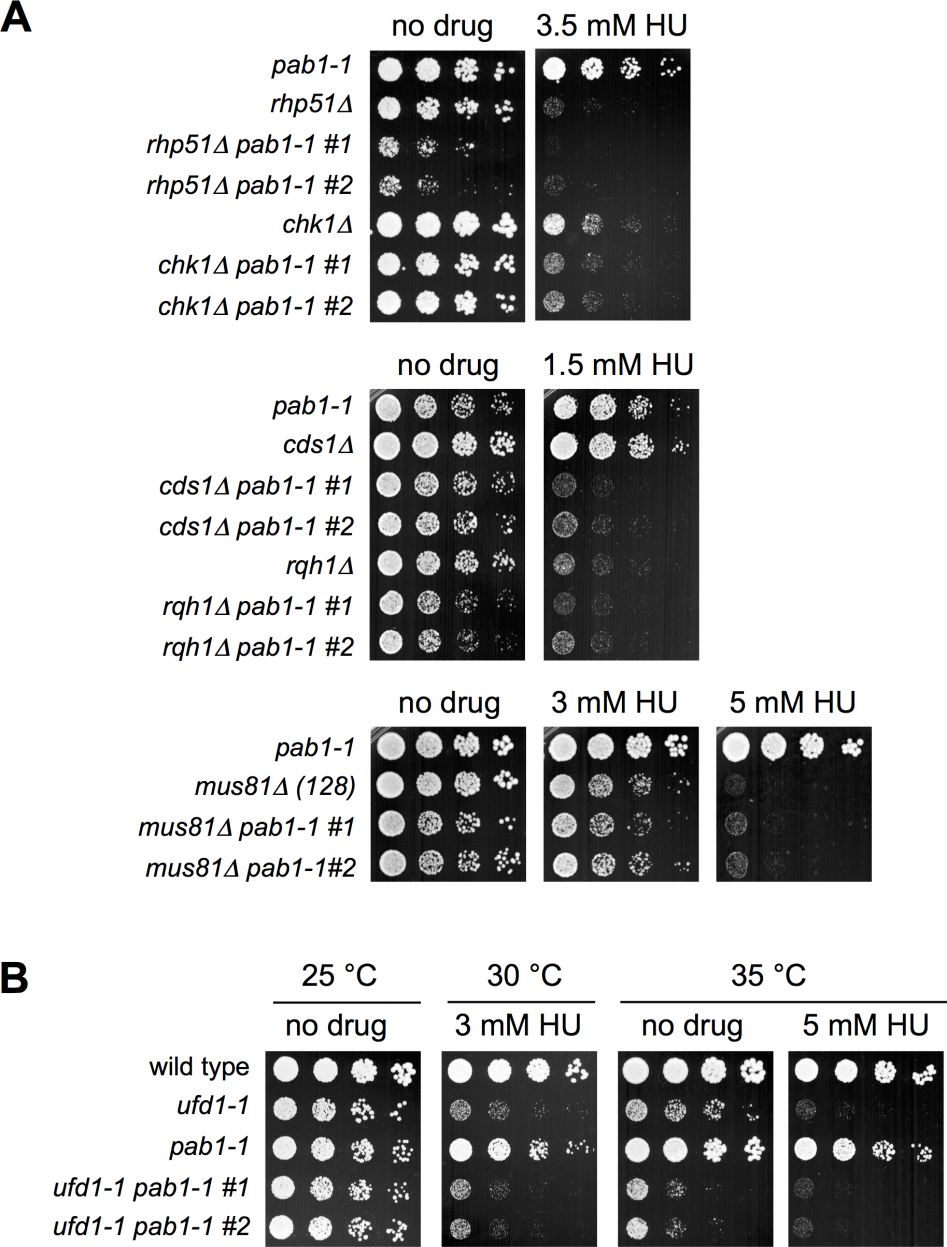
*Pab1-1* Is Not a General Suppressor of DNA Repair and Replication Stress Defects. *A* and *B*, dilution series of the indicated strains were spotted onto YES plates, with or without HU at the indicated concentrations and temperatures.

As PP2A is involved in a plethora of cellular functions, we next tested if *pab1-1* is a general suppressor of HU-induced replication stress and/or temperature sensitivity. Initially, we generated double mutants of *pab1-1* with either *chk1∆*, which lacks the G2 DNA damage checkpoint [29], or *rhp51∆* that compromises homologous recombination (HR) repair [30]. In contrast to the robust rescue of *slx8-29*, *chk1∆ pab1-1* double mutant cells showed additive sensitivity to HU (**Fig 3A**). Moreover, *rhp51∆ pab1-1* double mutant cells were synthetically sick in the absence of drug, and exhibited synergistic sensitivity to HU (**Fig 3A**). Next we tested if *pab1-1* can rescue *cds1∆* cells, which lack the replication checkpoint [31–33], or *rqh1∆* and *mus81∆* cells that exhibit severe HR defects under replication stress [34, 35]. Again, the double mutant strains were either synergistically sensitive to HU (e.g. *cds1∆ pab1-1*), or *pab1-1* provided no discernable growth benefit (**Fig 3A**). In addition, *pab1-1* does not rescue the temperature or HU sensitivity of an allele of the Cdc48/p97 cofactor Ufd1 (*ufd1-1*, **Fig 3B** [8]). Together, these data demonstrate that the rescue of *slx8-29* by *pab1-1* is not the result of a general increase in cellular resistance to genotoxins.

### Impact of CDK Activity and the Cell Cycle Checkpoints on the *slx8-29* Phenotype

**Fig 4:**
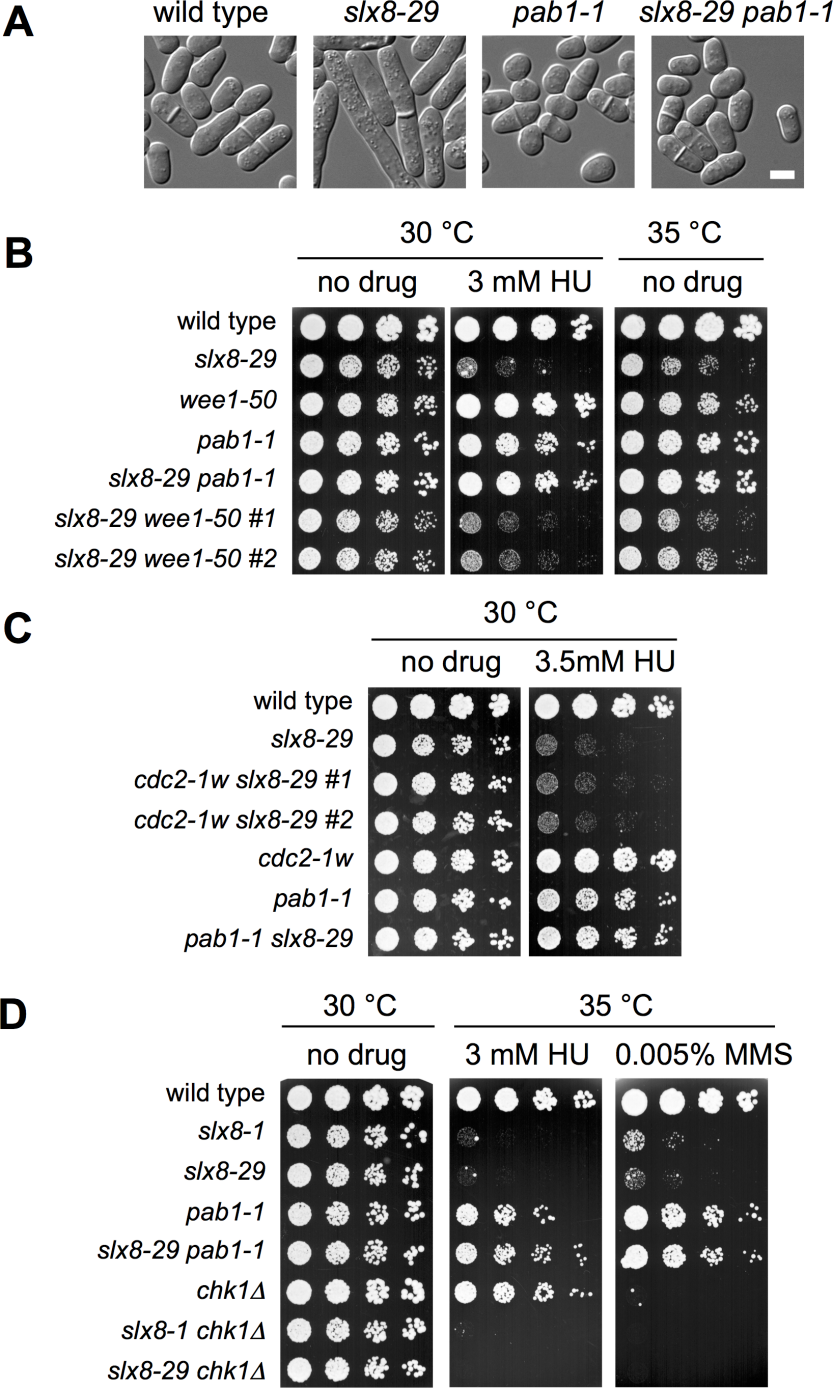
Impact of CDK Activity and the Cell Cycle Checkpoints on the *slx8-29* Phenotype. *A*, differential interference contrast (DIC) light microscopy of the indicated strains. *Bar*, 5 μM. *B-D*, dilution series of the indicated strains were spotted onto YES plates, with or without drug.

In fission yeast, *Xenopus* and mammalian cells, PP2A-Pab1^B55^ antagonizes CDK activity by regulating its activating phosphatase Cdc25 and inhibitory kinase Wee1 (see [36–38] & refs. therein). Consistent with this, *pab1-1* cells and those expressing lower than wild-type levels of Pab1 enter mitosis precociously at a reduced cell length (**Figs 4A** and **S1A** [38]). Therefore, we asked if elevated CDK activity *per se* mediates the rescue of *slx8-29* by *pab1-1*. To this end, we determined the genetic interactions between *slx8-29* and mutations that render CDK hyperactive or ablate the G2/M DNA damage checkpoint.

First we tested if a temperature sensitive Wee1 allele, *wee1-50*, was able to suppress *slx8-29*. *Wee1-50* cells, like *pab1-1*, enter mitosis at a reduced cell size due to hyperactive CDK [39]. However, *wee1-50* was unable to rescue the temperature and HU sensitivity of *slx8-29* (**Fig 4B**). As Wee1 could have functions beyond antagonizing CDK that obscure any rescue of *slx8-29*, we also tested if a hyperactive allele of CDK, *cdc2-1w* [40], was able to rescue *slx8-29*. Again, *cdc2-1w* could not restore growth to *slx8-29* at its restrictive temperature in the presence of HU, conditions in which *pab1-1* affords a near complete rescue (**Fig 4C**).

Finally, we tested if ablating the G2 DNA damage checkpoint, which normally inhibits CDK and the cell cycle following DNA damage, could rescue *slx8-29*. In the presence of HU at 35°C, conditions in which both *pab1-1* and *chk1∆* cells are fully viable, only *pab1-1* was able to rescue of *slx8-29* (**Fig 4D**). Moreover, *pab1-1* was able to rescue *slx8-29* cell growth in the presence of MMS, in addition to rescuing their temperature and HU sensitivity (**Fig 4D**).

Taken together, these data demonstrate that although advanced mitosis is one phenotypic consequence of *pab1-1*, as it is for mutants of the PP2A catalytic subunit Ppa2 [36], it is not the mechanism for *slx8-29* rescue.

### Impact of *pab1-1* on SUMO Pathway Activity

In fission yeast, reducing SUMO chain formation renders STUbL activity largely redundant [4]. For example, the SUMO^K14,30R^ or SUMO^D81R^ chain deficient mutants allow *slx8-29* cells to grow at high temperature, and in the presence of HU [4]. Therefore, we considered the possibility that the *pab1-1* mutation affects SUMO pathway activity and reduces SUMO chain production.

**Fig 5:**
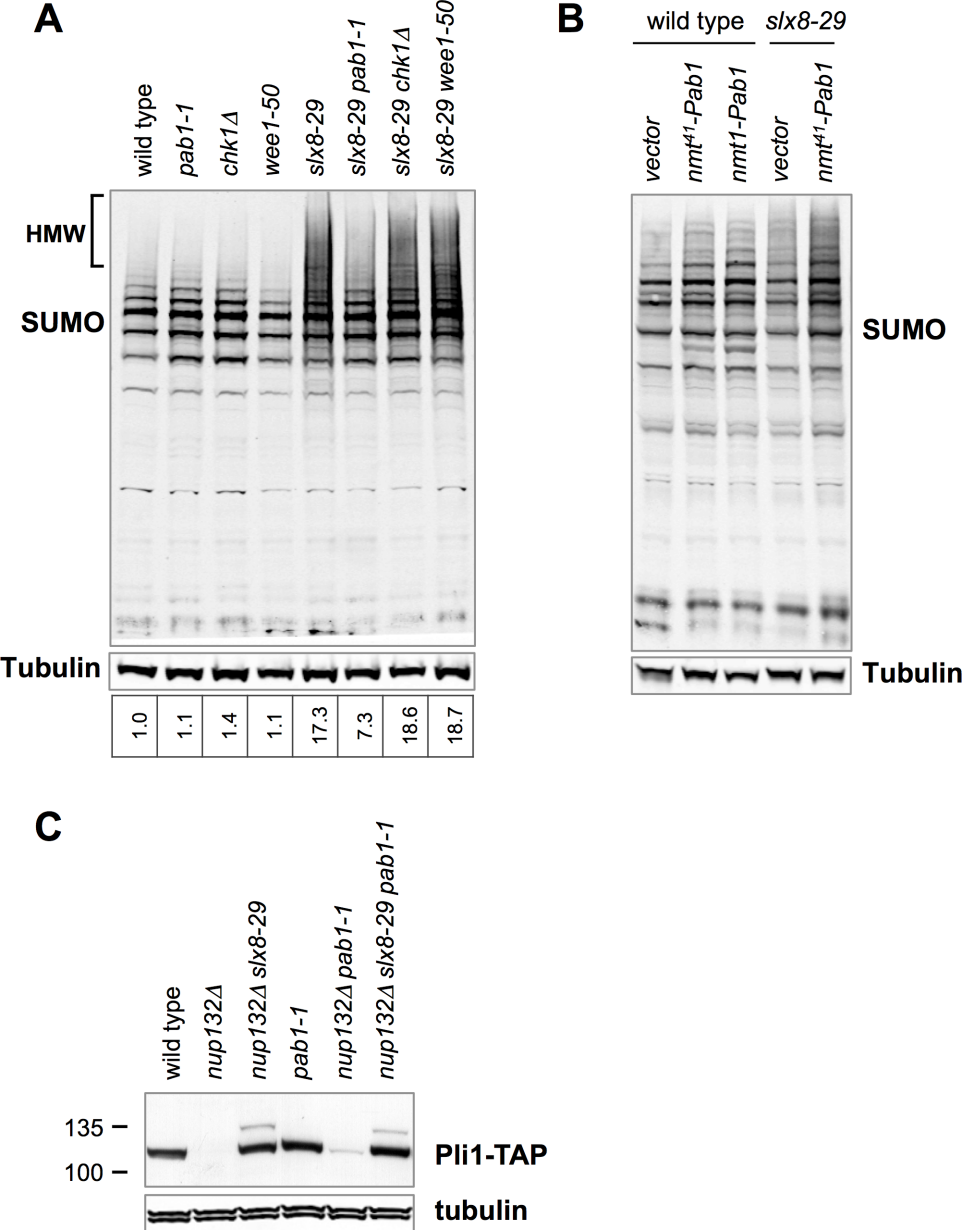
Impact of *pab1-1* on SUMO Pathway Activity. *A*, Western blots of sumoylated proteins or tubulin of indicated strains grown at 25°C to mid log phase then shifted to 35°C for 3 h. *B*, Western blots of sumoylated proteins or tubulin of indicated strains. Ectopic expression of Pab1 under the *nmt41* or *nmt1* promoter was induced by growing cells in the absence of thiamine (–B1) for 48 h at 25°C. *C*, Western blots of Pli1-TAP, detected by peroxidase anti-peroxidase (*PAP*), or tubulin of indicated strains grown at 25°C to log phase.

We compared SUMO conjugate levels in *slx8-29* single mutant cells versus double mutants of *slx8-29* with *pab1-1* and the controls *wee1-50* and *chk1∆*. The characteristic accumulation of high molecular weight (HMW) SUMO conjugates was observed in *slx8-29* single and *slx8-29 wee1-50* or *slx8-29 chk1∆* double mutants (**Fig 5A**). Although difficult to quantify due to their "smeary" nature, in contrast to *slx8-29* and the double mutant controls, HMW SUMO conjugates are reduced in *slx8-29 pab1-1* cells (**Fig 5A**). Because each of the *slx8-29 pab1-1*, *slx8-29 wee1-50* and *slx8-29 chk1∆* double mutants continue cycling, whereas *slx8-29* cells accumulate in G2, cell cycle position does not contribute significantly to the observed SUMO levels. Moreover, the observed HMW SUMO conjugate levels correlate well with the growth of each strain in the presence of HU i.e. reduced levels support *slx8-29* growth (**Fig 4**). Interestingly, ectopic expression of Pab1 in wild-type or *slx8-29* cells leads to a dose-dependent increase in HMW SUMO conjugates, which is more pronounced in *slx8-29* cells (**Fig 5B**). Because increased Pab1 dosage is toxic in *slx8-29* cells (**Fig 2**), there is again a good correlation between HMW SUMO conjugates and *slx8-29* cell viability.

As *slx8-29* is a temperature sensitive and not a null allele, *pab1-1* could conceivably "reactivate" it, leading to the observed reduction in HMW SUMO conjugates. We tested this possibility using a specific STUbL substrate we recently identified. In Nup132 mutant cells, the SUMO E3 ligase Pli1 is degraded in an Slx8-dependent manner [21]. Consistent with this, Pli1 is stabilized in Nup132 mutant cells by the *slx8-29* mutation (**Fig 5C**, [21]). Importantly, Pli1 stabilization by *slx8-29* is not reversed by *pab1-1* (*nup132∆ slx8-29 pab1-1*, **Fig 5C**). This result indicates that *pab1-1* does not reactivate the *slx8-29* mutant, but instead affects the accumulation of HMW SUMO conjugates either through the regulation of SUMO pathway factors, or activities that act in parallel to STUbL.

### Impact of *pab1-1* on SUMO Pathway Mutants Functionally Related to STUbL

Our data thus far indicate that *pab1-1* reduces the burden of SUMO chains in STUbL mutant cells, making them less dependent on this normally essential activity. We therefore tested the effect of *pab1-1* on other SUMO pathway mutants that experience SUMO chain-mediated toxicity.

**Fig 6:**
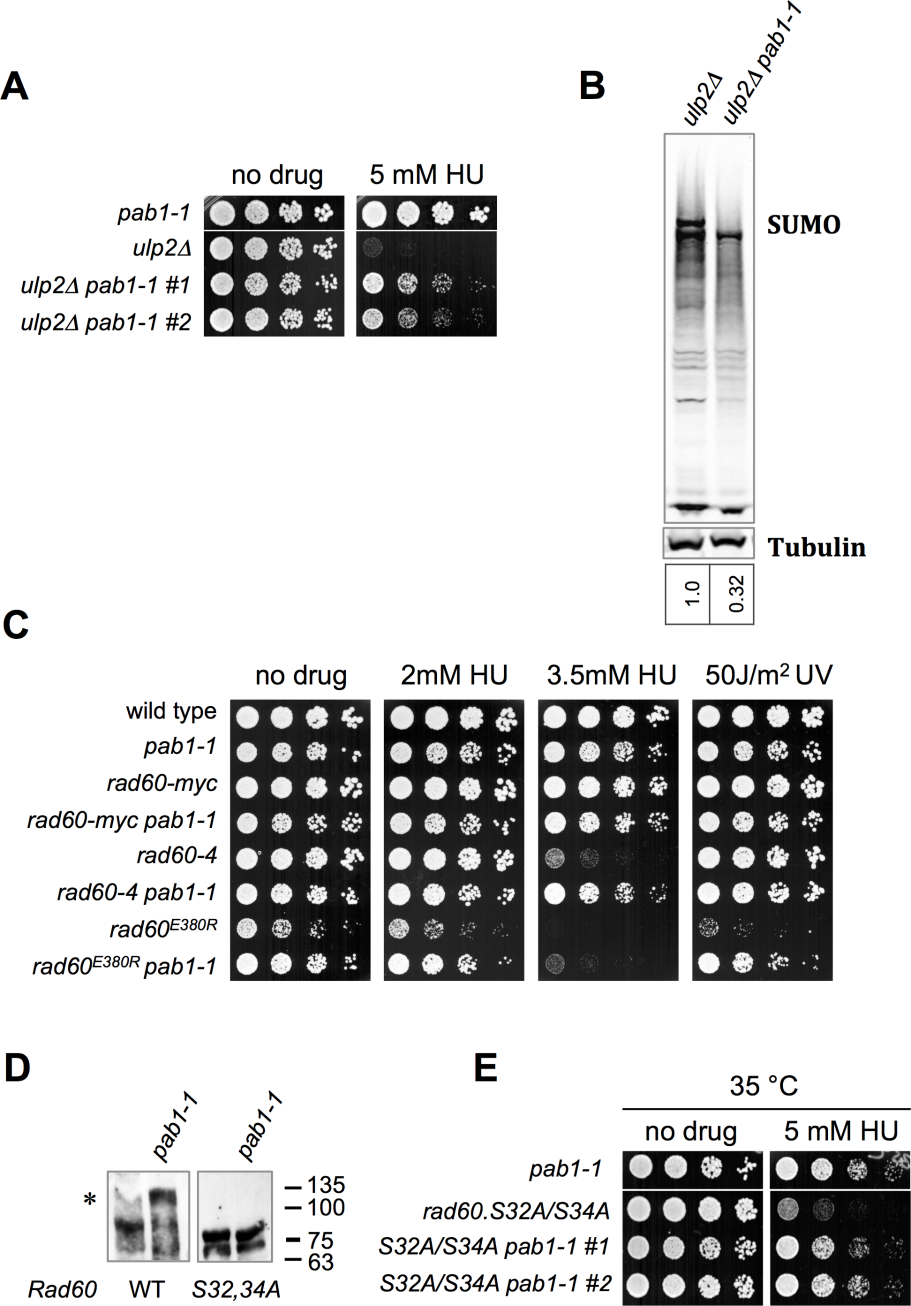
The *pab1-1* Mutation Rescues *ulp2* and *rad60* Mutants. *A*, C, *& E*, dilution series of the indicated strains were spotted onto YES plates, with or without the indicated genotoxic challenge. *B*, Western blots of sumoylated proteins or tubulin of indicated strains grown at 25°C to log phase. *D*, Western blotting of Myc-tagged Rad60 or Rad60.S32A/S34A mutant in wild type or *pab1-1* background using an antibody against Myc (9E10) and detected using ECL. Samples were run on 10% Phos-tag Tris-Glycine gel. Asterisk indicates phosphorylated Rad60 species.

In both fission and budding yeast Ulp2 SUMO protease mutants, SUMO chains accumulate and cause genome instability [4, 7]. This is evidenced by the suppression of *ulp2∆* phenotypes in the presence of a SUMO chain blocking mutant e.g. SUMO^K14,30R^ [4, 7]. Strikingly, as for *slx8-29*, we found that *pab1-1* strongly suppressed the HU sensitivity of *ulp2∆* cells (**Fig 6A**). Moreover, western analysis of SUMO again revealed a small but detectable reduction in HMW species in *ulp2∆ pab1-1* double mutant versus *ulp2∆* single mutant cells (**Fig 6B**).

The SUMO mimetic and genome stability factor Rad60 physically interacts with STUbL, and is functionally integrated with the SUMO pathway [4, 22, 41–44]. Rad60 contains two SUMO-like domains (SLD1 and SLD2) each of which interacts with different components of the SUMO pathway [4, 22, 43]. SLD1 interacts with STUbL, whereas SLD2 interacts with the SUMO conjugating enzyme Ubc9.

The SLD2:Ubc9 complex exists in competition with the SUMO:Ubc9 complex, as both use the same interface on Ubc9 [4, 43]. Because the SUMO:Ubc9 complex promotes SUMO chain formation *in vivo*, SUMO chains likely form in a *rad60*^*E380R*^ mutant that is unable to interact with Ubc9, due to an excess of the SUMO:Ubc9 complex [4]. Consistent with this, SUMO^K14,30R^ partially suppresses *rad60*^*E380R*^ HU sensitivity [4]. Interestingly, *pab1-1* also partially suppresses *rad60*^*E380R*^ sensitivity to HU and UV irradiation, and the HU sensitivity of another Rad60 mutant, *rad60-4* (**Fig 6C**). Together, these data indicate that *pab1-1* may mitigate SUMO-chain mediated toxicity in Ulp2 and Rad60 mutant cells, as it does in *slx8-29* cells. Moreover, as for *slx8-29*, the phenotypes of *rad60-4* and *ulp2∆* cells are not rescued by the hyperactive CDK allele *cdc2-1w*.

Given that Rad60 is phosphorylated during replication stress, and that it both physically and functionally associates with STUbL [20, 22, 41, 43, 44], we asked if it or other SUMO pathway factors were potential targets of PP2A-Pab1. To this end, we analyzed the phosphorylation status of Rad60, Slx8, Ubc9, and Pli1 in wild-type or *pab1-1* cells by comparing their migration on regular or phos-tag PAGE gels [45]. Amongst these proteins, only Rad60 phosphorylation was detectably enhanced by the *pab1-1* mutation (**Fig 6D)**. Strikingly, this Rad60 hyper-phosphorylation was abolished when putative casein kinase sites in Rad60, serine S32 and S34, were mutated to alanine (**Fig 6D**, [44]). As Rad60 controls SUMO pathway output [4, 43], this result raised the possibility that Rad60 phosphorylation might mediate the suppressive effects of *pab1-1* on *slx8-29*, *ulp2∆* and *rad60* mutants. However, *pab1-1* also rescues the HU sensitivity of *rad60*^*S32*,*34A*^, which is refractory to *pab1-1* induced phosphorylation (**Fig 6D and 6E**). Therefore, although *pab1-1* impacts Rad60 phosphorylation, it is not the critical or sole target of PP2A-Pab1.

### Effect of *pab1-1* on Chromosome Segregation in SUMO Pathway Mutant Cells

**Fig 7:**
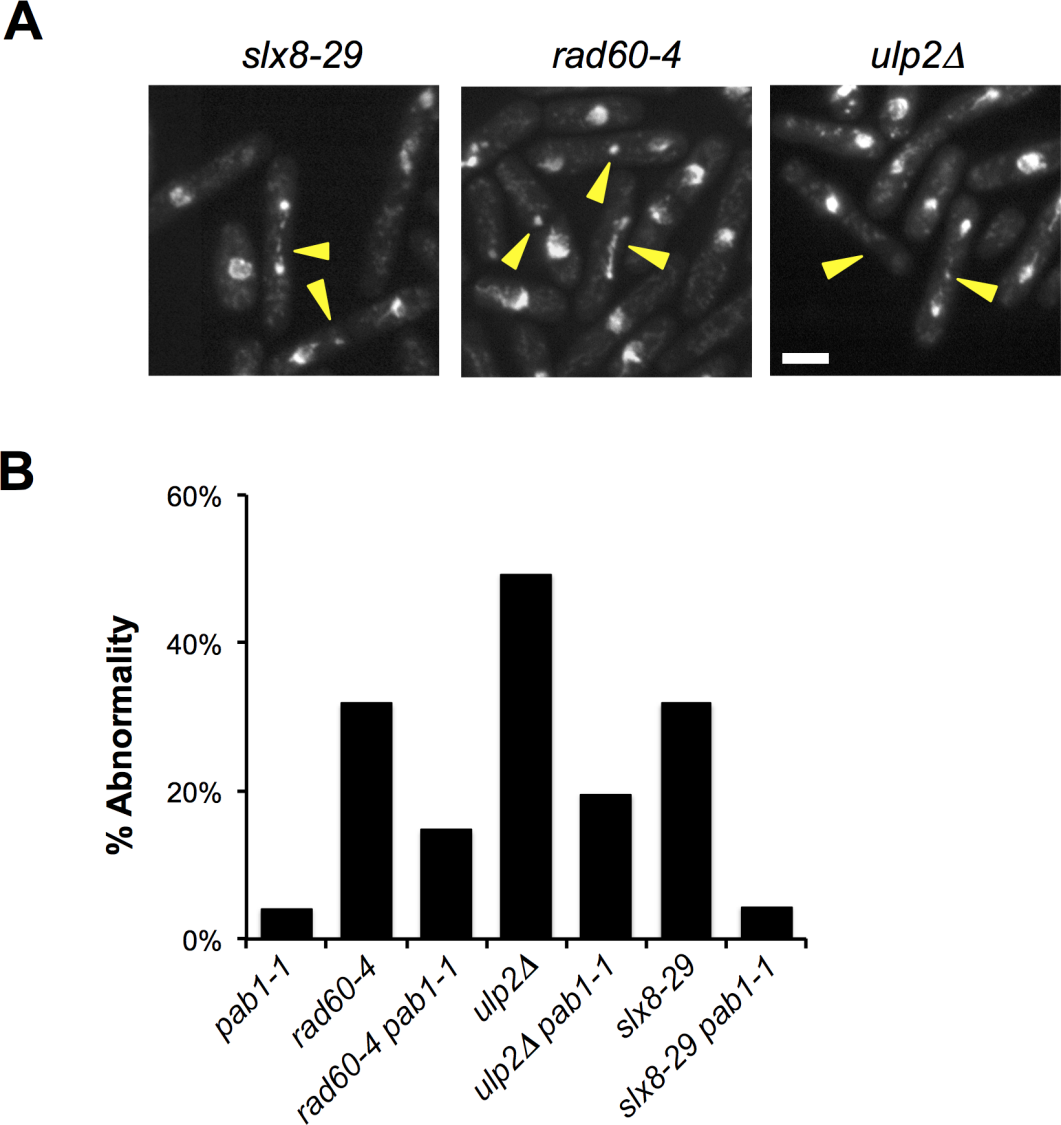
Effect of *pab1-1* on Chromosome Segregation in SUMO Pathway Mutant Cells. *A*, the indicated strains were grown in liquid YES medium at 30°C to early log phase before treatment with 15 mM hydroxyurea (HU) for 4–5 h to arrest cells in early S phase. At the end of the treatment, HU was washed out, and cells were allowed to grow in fresh YES for an additional 2 h before fixing, DAPI staining, and imaging. Yellow arrowheads mark examples of abnormal mitoses. *B*, the percentage of mitotic abnormalities quantified in binucleate cells (n ~100) of the indicated genotypes following release from HU arrest as in "*A*".

Homologous recombination (HR)-dependent chromosome linkages form that prevent normal chromosome segregation in *rad60* and *slx8* mutant cells after replication stress [22, 41, 42]. We therefore analyzed chromosome segregation in *slx8-29 pab1-1*, *slx8-29*, *rad60-4 pab1-1*, *rad60-4*, and *pab1-1* cells following release from HU-induced cell cycle arrest. After acute treatment, 4–5 hrs in 15 mM HU, *slx8-29* and *rad60-4* underwent abnormal mitoses in 32% and 31% of cells, respectively (**Fig 7**). In contrast, mitosis was aberrant in only 4% of *pab1-1* or *slx8-29 pab1-1* cells, and 14% of *rad60-4 pab1-1* cells (**Fig 7**). These data are consistent with the partial rescue of the chronic HU sensitivity of *slx8-29* and *rad60-4* by *pab1-1* (**Fig 6C**), and moreover, indicates that *pab1-1* suppresses aberrant HR in these mutants after replication stress.

Ulp2 mutant cells also undergo recombination-dependent mitotic catastrophe following replication stress, which can be suppressed by reducing SUMO chain formation [4, 7, 46]. We therefore tested the impact of *pab1-1* on chromosome segregation in *ulp2∆* cells following release from acute HU treatment. As for *rad60-4* and *slx8-29*, we observed increased mitotic abnormalities in *ulp2∆* (~50%), which were reduced (~19%) in *ulp2∆ pab1-1* cells (**Fig 7**). Again, this result mirrors the partial rescue of the HU sensitivity of *ulp2∆* cells by *pab1-1* (**Fig 6A**).

## Discussion

Sumoylation and phosphorylation are master regulators of cell growth and genome stability, with defects in the homeostasis of these key PTMs driving human disease. Accordingly, deregulation of PP2A, an abundant heterotrimeric serine/threonine phosphatase, is observed in many cancers [47]. Cancer associated mutations are found in each of the PP2A subunits: A (scaffold), B (regulatory/substrate targeting), and C (catalytic). Given its hub role in cell growth and tumorigenesis, PP2A is a therapeutic target of interest [47].

Likewise, the SUMO pathway and its regulators such as SENPs^ULPs^ and STUbL are therapeutically valuable in a number of cancers, including leukemia and those overexpressing the MYC oncogene [17, 18]. The widespread interest in PP2A and the SUMO pathway as therapeutic drug targets makes it important to identify potential compensatory mechanisms that could allow cells to escape such interventions.

Here, we reveal that a hypomorphic allele of a B subunit of fission yeast PP2A, Pab1^B55^, is a potent suppressor of the phenotypes of STUbL, Ulp2 and Rad60 mutants. Strikingly, in a separate screen for spontaneous suppressors of the HU sensitivity of *rad60*^*E380R*^ (to be reported elsewhere), we noted that some had a short cell phenotype, similar to that of *pab1-1*. Sequencing revealed these suppressors to be truncating mutations in the gene encoding Ypa2, an activator of fission yeast PP2A [28]. Together with the fact that *ypa2∆* also suppresses *slx8*-29 phenotypes (**Fig 2D)**, this result underscores the intriguing functional relationship between STUbL, Ulp2, Rad60 and PP2A. Evolutionary conservation of these factors suggests that such compensation could occur in human cells, undermining targeted therapies.

An overt phenotype of *pab1-1* cells, as for mutations in certain other PP2A subunits, is their entry into mitosis at a reduced cell size due to elevated CDK activity (**Figs 4A** and **S1A;** [36–38] & refs. therein)). Defects in the SUMO pathway have previously been linked to a failure of cells to reenter the cell cycle after completion of DNA repair e.g. [48, 49]. Therefore, we initially speculated that the suppressive effects of *pab1-1* were due to elevated CDK activity, and eventual bypass of the checkpoint induced cell cycle arrest. Indeed, the sickness of *pab1-1* when combined with DNA repair mutants such as *rhp51∆* and *rqh1∆* is consistent with an attenuated G2/M cell cycle checkpoint, as in *chk1∆* cells [50]. Moreover, the synthetic sickness of *pab1-1* and *cds1∆* in the presence of HU mirrors that of *cds1∆ chk1∆* double mutants, which fail to delay the cell cycle in response to replication inhibition (**Fig 3A**, [31]).

However, neither hyperactive CDK (e.g. *cdc2-1w*, *wee1-50*), nor ablating the damage checkpoint (*chk1∆*) suppresses STUbL dysfunction. This specificity is all the more surprising given that *cdc2-1w* shares several phenotypes with *pab1-1*, including small cell size and sensitivity to CPT [51]. Therefore, *pab1-1* likely affects the phosphorylation status of another kinase target, which in turn reduces cellular dependency on STUbL activity.

Others and we have shown that reducing SUMO chain formation bypasses the need for the SUMO protease Ulp2 [4, 7]. Moreover, the functions of STUbL can be bypassed by blocking SUMO chain formation in fission yeast [4, 13]. Western analysis of STUbL and Ulp2 mutants revealed that the *pab1-1* allele reduces the abundance of HMW SUMO conjugates in both backgrounds, whereas overproducing Pab1 causes an increase. This effect is not due to cell cycle position, indicating that PP2A-Pab1^B55^ either directly or indirectly modulates SUMO pathway homeostasis.

SUMO and its E3 ligases are subject to phosphorylation-dependent regulation, providing a potential avenue for direct PP2A-dependent regulation [52–54]. However, we were unable to detect PP2A-Pab1 regulated phosphorylation of any SUMO pathway factors, other than Rad60. In addition, Rad60 hyper-phosphorylation was excluded as a contributor to the suppression of *slx8-29*, *ulp2∆* or *rad60* mutants by *pab1-1*. This illustrates the difficulty in identifying one amongst a plethora of cellular targets of PP2A-Pab1 that mediates the effects of *pab1-1* on the SUMO pathway. This is certainly a goal for the future, and may be assisted by more global approaches such as phosphoproteome analysis of wild-type and *pab1-1* cells. In this regard, a recent study compared global protein phosphorylation levels between wild-type and *pab1∆* cells [55]. Unfortunately, amongst the numerous phosphoproteins identified, none were obvious candidates for mediators of the *pab1-1* phenotypes revealed here. This study also highlighted a limitation with such proteome-wide analyses, wherein the data are “swamped” by the most abundant PP2A-Pab1 targets such as metabolic enzymes. These may mask low abundance factors that are critical for the *pab1-1* SUMO-related phenotypes.

We also considered the possibility that the PP2A-Pab1^B55^ enzyme is a target of STUbL, Ulp2 and Rad60, and that *pab1-1* bypasses the requirement for such regulation (see below). Indeed, in budding yeast, STUbL activity prevents the kinetochore proximal accumulation of a distinct PP2A complex, PP2A-Rts1^B56^ [56]. How STUbL antagonizes PP2A-Rts1^B56^ accumulation was not determined, but it was suggested to be through an indirect mechanism involving activation of the tension-sensing pathway, leading to increased Rts1^B56^ recruitment via Sgo1 [56]. Nevertheless, the positive genetic interaction between STUbL and PP2A-Rts1^B56^ raises interesting questions about the crosstalk between the SUMO and PP2A pathways across species. For example, does Rts1^B56^ mutation suppress budding yeast Ulp2 and Esc2 (Rad60 orthologue) mutants? If so, this could indicate that the SUMO-related functions of PP2A rely on distinct complexes in different organisms i.e. PP2A-Rts1^B56^ in budding yeast or PP2A-Pab1^B55^ in fission yeast.

To test PP2A-Pab1^B55^ as a target of the SUMO pathway, we analyzed Pab1 protein levels, potential SUMOylation state, and subcellular localization in *slx8-29* and *ulp2∆* cells, but observed no significant differences (**S1B and S1C Fig**). In addition, two recent proteomic analyses of protein SUMOylation in fission yeast failed to detect modification of any of the abundant PP2A complex proteins, even in cells with compromised STUbL activity [57, 58]. Although we cannot completely exclude direct or indirect regulation of PP2A-Pab1^B55^ by the SUMO pathway, we favor a model in which the hyper-phosphorylation of PP2A-Pab1^B55^ targets in *pab1-1* cells mitigates the phenotypes of the tested SUMO pathway mutants.

For example, in *pab1-1* cells, a pathway parallel to STUbL that degrades HMW SUMO species could be engaged e.g. through a phosphodegron in key SUMO conjugates. However, because *pab1-1* suppresses *slx8-29*, *ulp2∆* and *rad60* phenotypes, this would imply the existence of a single or an overlapping set of SUMO conjugates that cause the phenotypes of each mutant. Although possible, it seems more likely that PP2A-Pab1^B55^ reduces the SUMO chain output of the SUMO pathway, thus providing pan-suppression of STUbL, Ulp2 and Rad60 mutants.

Replication stress induces lethal HR-dependent chromosome missegregation in STUbL, Rad60 and Ulp2 mutant cells [20, 22, 42, 46]. We found that in keeping with the ability of *pab1-1* to improve the growth of each mutant in the chronic presence of HU, *pab1-1* reduces HU-induced mitotic chromosome missegregation in the mutants. As SUMO chains drive the increased mitotic recombination and chromosome missegregation in *ulp2∆* budding yeast [46], our chromosome segregation analyses of *slx8*-29, *rad60-4* and *ulp2∆* are again consistent with a role for PP2A-Pab1^B55^ in controlling SUMO pathway homeostasis.

Overall, we have identified critical functional crosstalk between the SUMO pathway and the major serine/threonine phosphatase PP2A-Pab1^B55^. Interplay between these master regulators of cell growth and genome stability is intrinsically important, and in the future will likely provide a basis for mechanistically defining phosphorylation-dependent regulation of SUMO pathway output.

## Materials and Methods

### General Yeast Techniques

Standard methods for *S*. *pombe* were performed as described previously [59]. All strains (Table 1) are of genotype *ura4-D18 leu1-32* unless otherwise stated.

**Table 1:** List of yeast strains used in this study.

Strain	Genotype*	Source
NBY780	*h*^*+*^	
NBY781	*h*_*¯*_	
PR298	*wee1-50*	
PR561	*cdc2-1w*	
NBY28	*cds1*::*ura4*^*+*^	
NBY128	*mus81*::*kanMx6*	
NBY202	*rqh1*::*ura4*^*+*^	
NBY203	*rad60-myc*:*kanMx6*	
NBY303	*rad60-4*:*kanMx6*	
NBY952	*rhp51*::*ura4*^*+*^	
NBY1008	*slx8-1*:*kanMx6*	
NBY1097	*rad60[S32/A*, *S34/A]-myc*:*kanMx3*	
NBY1111	*chk1*::*ura4*^*+*^	
NBY1159	*slx8-1*:*kanMx6 chk1*::*ura4*^*+*^	
NBY1820	*pli1-TAP*:*kanMx6*	
NBY2078	*rad60*^*E380R*^:*kanMx6*	
NBY2471	*slx8-29*:*kanMx6*	
NBY2756	*slx8-29*:*kanMx6* pREP2:*ura4*^*+*^ integrated at *ars1*	
NBY2936	*slx8-29*:*kanMx6 chk1*::*ura4*^*+*^	
NBY2957	*rad60*^*E380R*^:*kanMx6 pab1-1*	
NBY2983	*pab1-1 rhp51*::*ura4*^*+*^	
NBY2984	*pab1-1 chk1*::*ura4*^*+*^	
NBY3418	pREP41:*LEU2*	
NBY3420	*slx8-29*:*kanMx6* pREP41:*LEU2*	
NBY3566	*slx8-29*:*kanMx6*	
NBY3567	*slx8-29*:*hphMx6*	
NBY3568	*ulp2*::*kanMx6*	
NBY3918	*ufd1-1*:*kanMx6*	
NBY5022	*pab1-1*	
NBY5027	*slx8-29*:*kanMx6 pab1-1*	
NBY5028	*ulp2*::*kanMx6 pab1-1*	
NBY5055	*pab1-1 cds1*::*ura4*^*+*^	
NBY5056	*pab1-1 mus81*::*kanMx6*	
NBY5057	*pab1-1 rqh1*::*ura4*^*+*^	
NBY5069	*pab1-1 ufd1-1*:*kanMx6*	
NBY5109	*slx8-29*:*kanMx6 wee1-50*	
NBY5135	*tetO*_*7*_*-TATA*_*CYC1*_*-FLAG*_*3*_*-pab1*:*kanMx6*	
NBY5137	*tetO*_*7*_*-TATA*_*CYC1*_*-FLAG*_*3*_*-pab1-1*:*kanMx6*	
NBY5139	pJK148-*nmt41x-GFP-pab1*:*leu1*^*+*^ integrated at *leu1*	[25]
NBY5141	*pab1*::*kanMx6*	[25]
NBY5151	pJK148-*nmt41x-GFP-pab1*:*leu1*^*+*^ integrated at *leu1 slx8-29*:*hphMx6*	
NBY5184	pREP41-*pab1*:*LEU2*	
NBY5193	*slx8-29*:*hphMx6 pab1*::*kanMx6*	
NBY5195	*tetO*_*7*_*-TATA*_*CYC1*_*-FLAG*_*3*_*-pab1*:*kanMx6 slx8-29*:*hphMx6 pDM291–tetR–tup11Δ70*:*ura4*^*+*^	
NBY5196	*tetO*_*7*_*-TATA*_*CYC1*_*-FLAG*_*3*_*-pab1*:*kanMx6 slx8-29*:*hphMx6*	
NBY5217	*slx8-29*:*kanMx6* pREP41-*pab1*:*LEU2*	
NBY5332	pREP1-*pab1*:*LEU2*	
NBY5336	*rfp1*::*kanMx6 pab1-1*	
NBY5337	*rfp2*::*hphMx6 pab1-1*	
NBY5343	*rfp1*::*kanMx6 rfp2*::*hphMx6 pab1-1*	
NBY5354	*pab1-HA*:*hphMx6* pREP41-*H*_*6*_*-SUMO*:*LEU2*	
NBY5361	*pab1-HA*:*hphMx6 ulp2*::*kanMx6*	
NBY5362	*pab1-HA*:*hphMx6 slx8-29*:*kanMx6*	
NBY5391	*slx8-29*:*kanMx6 cdc2-1w*	
NBY5487	*ypa2*::*ura4*^*+*^	
NBY5489	*ppa2*::*ura4*^*+*^	
NBY5504	*ypa2*::*ura4*^*+*^ *slx8-29*:*kanMx6*	
NBY5505	*ppa2*::*ura4*^*+*^ *slx8-29*:*kanMx6*	
NBY5506	*pli1-TAP*:*kanMx6 nup132*::*ura4*^*+*^	
NBY5546	*pli1-TAP*:*kanMx6 nup132*::*ura4*^*+*^ *slx8-29*:*hphMx6*	
NBY5574	*pli1-TAP*:*kanMx6 pab1-1*	
NBY5672	*pli1-TAP*:*kanMx6 nup132*::*ura4*^*+*^ *slx8-29*:*hphMx6 pab1-1*	
NBY5673	*pli1-TAP*:*kanMx6 nup132*::*ura4*^*+*^ *pab1-1*	
NBY5744	*rad60-myc*:*kanMx6 pab1-1*	
NBY5745	*rad60-4*:*kanMx6 pab1-1*	
NBY5834	*rad60[S32/A*, *S34/A]-myc*:*kanMx3 pab1-1*	
NBY5869	*pab1-1 cdc2-33*	

* All strains are of *ura4-D18 leu1-32* background genotype, unless otherwise stated. Double colons represent knockouts; single colons represent tagging. A reference is given for strains not generated in this study.

### Spot Assays

Cells were grown at 25°C to logarithmic phase (optical density at 600 nm [OD_600_] of 0.6 to 0.8), spotted in 5-fold dilutions from a starting OD_600_ of 0.5 on plates supplemented with the relevant drug. The plates were then incubated at 25 to 35°C for 3 to 5 days.

### Microscopy

For live imaging, cells were grown in liquid EMM medium supplemented with leucine, uracil, arginine, and histidine (LUAH) and sterilized by filtration to logarithmic phase. Wide-field images of live cells were acquired using a Nikon Eclipse microscope with a 100x Plan Apochromat DIC H oil immersion objective and a Photometrics Quantix charge-coupled device camera. Images were analyzed with NIH ImageJ software. For staining with 4',6-diamidino-2-phenylindole (DAPI), cells were fixed for 5 minutes in 70% ethanol (EtOH), washed in PBS, and resuspended in 250 ng/ml of DAPI prior to imaging.

### FLAG-IP and Western Blotting

To immunoprecipitate N-terminally FLAG-tagged wild type or mutant Pab1, cell pellets were resuspended in 0.4 ml of Sp-lysis buffer (50 mM Tris, pH 8, 150 mM NaCl, 1 mM EDTA, 10% glycerol, 0.1% Nonidet P-40) [8], supplemented with 2 mM PMSF, and Complete protease inhibitor tablet, EDTA-free (Roche Applied Science), and lysed by beating with silica–zirconia beads three times at 5.0 m/s for 20 s in a FastPrep-24. After 10 min clarification by centrifugation at 16,000 x g in a microfuge at 4°C, supernatant was quantified for protein concentration based on OD reading at 280 nm. A total of 1 mg of proteins were incubated with 20 μl of Protein G MagBeads (GenScript) that have been preequilibrated with 10 μg of mouse-anti-FLAG antibodies (M2, Sigma). After 1 h of binding at 4°C, the beads were washed extensively with Sp-lysis buffer, and eluted with 2x LDS Sample Loading buffer (Life Technologies). One fifth of the eluted proteins, along with 10 μg (1%) of the input, were separated by SDS-PAGE. Western blotting was carried out as previously described [8, 58]. The membrane was blocked in 1% *w/v* non-fat milk in phosphate buffer saline solution with 0.1% *v/v* Tween-20, probed with primary antibodies, followed by HRP or IRDye-conjugated secondary antibodies, and detected either using an ECL Dura system (Pierce) on film; or scanning on an ODYSSEY scanner (Li-Cor). TAP-tagged proteins were probed with Peroxidase-Antiperoxidase (PAP), then directly detected using ECL.

Protein phosphorylation was identified by comparing the migration of a protein species on 10% Tris-Glycine gel (Life Technologies) to that on 10% SuperSep Phos-tag (50 μmol/L, Wako) [45].

### Genome Sequencing Using Next Generation Sequencing

To extract genomic DNA, 10 ml of saturated cultures were collected by centrifugation and washed with 0.5 ml of H_2_O. The cell pellet was resuspended in 0.2 ml of extraction buffer (0.2% Triton X-100, 1% SDS, 100 mM NaCl, 10 mM Tris-HCl, pH 8.0) and transferred to a screw cap 1.5 ml tube in which 0.2 ml of PCIA (phenol:chloroform:isoamyl alcohol at a ratio of 25:24:1) has been added. The cells were lysed by bead-beating as described above. Afterward, the lysate was centrifuged for 5 min at 16,000 x g in a microfuge, and the upper layer was transferred to a fresh tube and extracted twice with chloroform. The aqueous layer was digested with 50 μg of RNase A for 30 min at 37°C, then extracted again with PCIA, followed with two chloroform extractions. The genomic DNA was precipitated with 3 M sodium acetate and 100% ethanol. The DNA pellet was washed with 70% ethanol, dried and dissolved in 80 μl of TE (10 mM Tris, pH 8.0, 1 mM EDTA).

For Next Generation Sequencing, 1 μg of genomic DNA was sheared on an S2 Covaris set at 10% duty cycle, intensity of 5, 200 cycles per burst for 120 seconds, to obtain fragments of 200–300 bp in sizes. The fragments were then end-repaired, A-tailed with Taq Polymerase, kinased, and ligated to standard TruSeq (Illumina) barcoded adapters following manufacturer recommended protocols. The library was then amplified by PCR for 6 cycles. The amplified libraries were gel purified to select for DNA products of between 200–250 bp for single read 1x100 sequencing. The 100bp reads were generated by the HISeq 2000 Analyzer (Illumina) located at the Scripps DNA Sequencing Facility. The Genome Analyzer Pipeline Software (currently Casava v1.8.2) was used to perform the early data analysis of a sequencing run, using tools including image analysis, base calling, and demultiplexing. The reads were aligned to the genome with Bowtie 0.12.9 software using parameter to keep only the best scoring singleton.

### Plasmids Construction

To overexpress the *pab1* gene, the *pab1* coding sequence was amplified from a cDNA library using the primer pair Omn330 and Omn331 (Omn330: 5’-AGATTCATATGGATGATATAGAAGACTCTTTGGATC-3’; Omn331: 5’-GACTAGGATCCTTAGAGCTTAGAGAAAACAAAAAGATTATTAG-3’), and cloned into pREP41 and pREP1 plasmids at the NdeI and BamHI sites, to generate pREP41-pab1 and pREP1-pab1.

## Supporting Information

S1 Fig *A*, The percentage of septated cells was monitored following a block at 35.5°C for 4 hrs and release to 25°C of *cdc2-33* and *cdc2-33 pab1-1* cells. The data shows that the *pab1-1* mutation promotes cell cycle progression, as anticipated. *B*, Western analysis of HA-tagged Pab1 shows similar expression of Pab1 in cells overexpressing His6-SUMO (-B1), deleted of *ulp2*, or with the *slx8-29* mutation. *C*, GFP-Pab1 shows similar nuclear/cytoplasmic distribution in wild type and *slx8-29* cells.
(TIF)
