# Supplementary figures and images for: Functional Crosstalk between the PP2A and SUMO Pathways Revealed by Analysis of STUbL Suppressor, razor 1-1
Source: PLoS Genet. 2016 Jul 11;12(7):e1006165. doi: 10.1371/journal.pgen.1006165 (PMC4939958; doi:10.1371/journal.pgen.1006165)

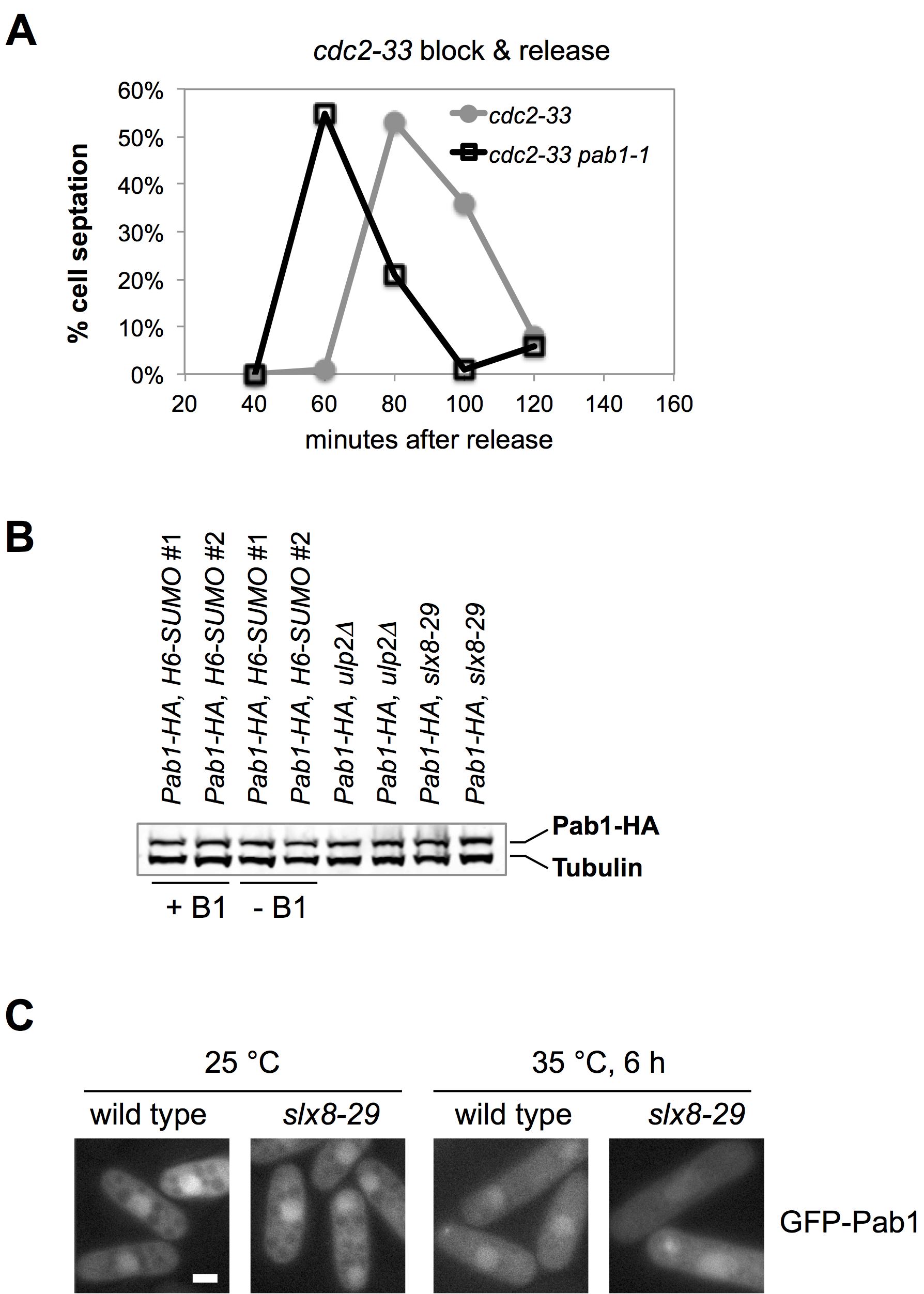

Supplement: S1 Fig — A, The percentage of septated cells was monitored following a block at 35.5°C for 4 hrs and release to 25°C of cdc2-33 and cdc2-33 pab1-1 cells. The data shows that the pab1-1 mutation promotes cell cycle progression, as anticipated. B, Western analysis of HA-tagged Pab1 shows similar expression of Pab1 in cells overexpressing His6-SUMO (-B1), deleted of ulp2, or with the slx8-29 mutation. C, GFP-Pab1 shows similar nuclear/cytoplasmic distribution in wild type and slx8-29 cells. (TIF) [file pgen.1006165.s001.tif]
